# Proteomic Solutions for Analytical Challenges Associated With Alcohol Research

**Published:** 2008

**Authors:** Michael J. MacCoss, Christine C. Wu

**Keywords:** Alcohol dependence, genetic theory of alcohol and other drug use, genetic trait, brain, animal models, proteins, protein analysis, proteomics, mass spectrometry, peptides

Alcohol addiction is a complex disease with both hereditary and environmental influences. Because molecular determinants contributing to this phenotype are difficult to study in humans, numerous rodent models and conditioning paradigms^1^ have provided powerful tools to study the molecular complexities underlying these behavioral phenotypes (Crabbe 2002). In particular, specifically bred rodents (i.e., selected lines and inbred strains) that differ in voluntary alcohol drinking represent valuable tools to dissect the genetic components of alcoholism. However, because each model has distinct advantages, a combined comparison across datasets of different models for common changes in gene expression would provide more statistical power to detect reliable changes as opposed to the analysis of any one model. Indeed, meta-analyses of diverse gene expression datasets were recently performed to uncover genes related to the predisposition for a high alcohol intake. This large endeavor resulted in the identification of 3,800 unique genes that significantly and consistently changed between all included mouse lines and strains (Mulligan et al. 2006).

Similar experiments also are crucial at the protein level. However, these analyses are not yet possible. Proteins do not conform to any one uniform sample preparation method and/or biochemical analysis. They display a broad range of physical and chemical properties (e.g., molecular weight or hydrophobicity) and are expressed over a very large dynamic range (up to 8 orders of magnitude) (Anderson 2005; Ghaemmaghami et al. 2003). Further complicating global proteomic comparisons are the added considerations that proteins often undergo extensive covalent modifications and that protein functions often are regulated by complex protein–protein interactions and the specific location of the proteins in the cell (i.e., their sub-cellular localization) (Grant and Wu 2007). Furthermore, because the number of biological replicates involved in behavioral analyses typically is high, robust high-throughput proteomic platforms will be required to handle the multitude of protein samples that can potentially result from the various brain regions for the numerous animal models and paradigms. Finally, these effects often are monitored over time courses, again inflating the total number of samples that need to be analyzed and compared. This article summarizes some general strategies for large-scale, high-throughput protein analyses and describes two new proteomic strategies that appear promising for future studies in this field.

## General Strategies of Large-Scale Protein Analyses

To overcome the complexities of proteins, proteomic methods routinely digest proteins into smaller pieces (i.e., peptides) prior to analysis. (Strategies using this approach are collectively termed shotgun proteomics.) The peptides then are separated by microcapillary chromatography and electro-sprayed directly into a mass spectrometer placed at the outlet of the chromatography column for mass analysis and fragmentation of the peptides^2^ using a technique known as data-dependent acquisition. In data-dependent acquisition, the mass spectrometer software makes real-time decisions about how the mass spectrometer is scanned, depending on data acquired in prior scans. Thus, the mass spectrometer acquires a “survey” mass spectrum, followed by tandem mass spectra (MS/MS spectra) of precursor ions identified in the earlier survey mass spectrum. This data-dependent acquisition, although powerful, is normally focused on the most intense signals and will ultimately be limited by the complexity of the mixture and the scan speed of the mass spectrometer.

The mass spectrometer’s ability to handle extremely complex mixtures of peptides can be enhanced either by biochemically fractionating the protein sample prior to digestion (thereby reducing the complexity of each resulting fractionated protein sample) or by adding an extra dimension of liquid chromatography (thereby increasing the separation on the peptide level) prior to mass spectrometry (Peng et al. 2003; Washburn et al 2001; Wolters et al. 2001). By improving peptide separation, the complexity of the set of peptides entering the mass spectrometer at each point in time is reduced and the mass spectrometer has more time to sample peptides eluting from the column with reduced interference from higher abundance peptide species. These multidimensional separation methods interfaced with fast-scanning mass spectrometers^3^ (Blackler et al. 2006; Schwartz et al. 2002) are very powerful and have improved researchers’ ability to identify large numbers of proteins from complex mixtures. However, this chromatographic separation and biochemical fractionation is slow, often resulting in an analysis time of greater than 24 hours per sample (MacCoss et al. 2002; Washburn et al. 2001). These long analysis times make analyses of replicate samples, multiple conditions, and many time points prohibitive. As a result, multidimensional chromatography essentially is incompatible with the throughput required for proteomic analysis of large numbers of samples (such as those commonly acquired in a behavior study). Furthermore, the comparison of peptides between samples using this approach is complicated, as proteins expressed in low abundance rarely are sampled because of the semirandom sampling of spectra by data-dependent acquisition (Liu et al. 2004).

An alternative to multidimensional chromatography is to use different mass spectrometers in distinct ways to improve the handling of complex mixtures (Conrads et al. 2000; Hu et al. 2005; Syka et al. 2004). Recent advances in mass spectrometry have facilitated the routine acquisition of mass spectra at high resolution with sufficient resolution and peak capacity to handle the complexity of the mixture with only a single dimension of chromatographic separation. Furthermore, proteomics researchers are beginning to adopt mass spectrometry–based approaches originally developed for small molecule quantitation workflows for the targeted analysis of hypothesized proteins (Anderson and Hunter 2006; Barnidge et al. 2004; Gerber et al. 2003). These advances in proteomic technologies provide a glimpse of future possibilities and applications toward the dissection of the molecular mechanisms of behavior. The following sections focus on two emerging technologies that look extremely promising for large-scale shotgun proteomic endeavors: label-free differential mass spectrometry for discovery-based comparative proteomics and targeted mass spectrometry for hypothesis-driven quantitative proteomics.

## Label-Free Differential Mass Spectrometry for Comparative Proteomics (Discovery Platform)

Most proteomics methods have been developed to acquire MS/MS spectra on as many molecular species as possible, regardless of whether those peptides are of interest. This semirandom sampling of peptides by data-dependent acquisition focuses on the most abundant peptides first and then samples lower intensity peptides in subsequent scan events. Although improvements in mass spectrometer scan speeds have increased the depth to which peptides can be sampled (Blackler et al. 2006; Mayya et al. 2005), this approach routinely wastes the majority of instrument time on sampling the most abundant molecular species in the sample.

**Figure 9:**
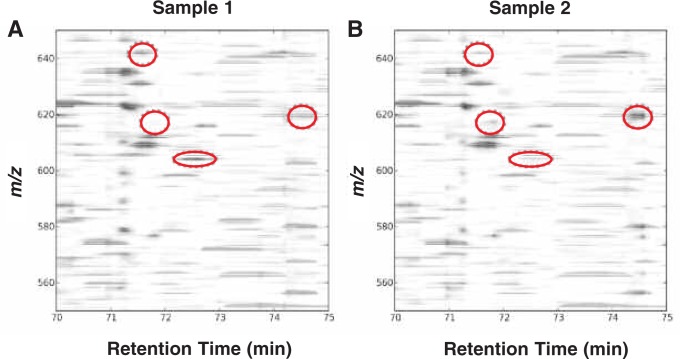
Finding differences between samples using differential mass spectrometry (dMS). Peptide maps are plotted as two-dimensional images following chromatogram alignment and intensity normalization. Statistical analysis software is used to find regions of mass-to-charge ratio (*m/z*) and retention time that differ in abundance between sample groups. Obvious visual differences (in the context of this figure) are illustrated in red.

Because of this limitation, some groups have begun to focus their identification efforts only on peptides that differ in abundance between samples (Finney et al. 2008; Pasa-Tolic et al. 2002; Prakash et al. 2006; Wiener et al. 2004). This experimental workflow is similar to that used in a two-dimensional (2D) gel analysis,^4^ except that these analyses are performed on the peptide level using chromatographic separation and not on the protein level using gel electrophoresis. Specifically, in a 2D gel experiment, gels are run first to identify spots that differ between samples and then efforts are taken to determine the identities of the proteins in those spots. Similarly, in a “differential” mass spectrometry experiment, chromatographic separations are first carried out to identify peaks that differ in abundance between samples (see figure 9) and then efforts are taken to determine the identities of the peptides in those peaks. Peak abundances that change in a statistically meaningful way (see figure 9, red circles) then are analyzed again to assign peptide identities.

The key experimental components to this differential mass spectrometry approach are (1) reproducible sample preparation, (2) an instrument that has sufficient peak capacity and dynamic range to handle complex mixtures with short analysis times, and (3) software for detecting regions of mass-to-charge ratio (*m/z*) and time that differ in abundance between samples. The mass chromatograms between experiments are first aligned, and subsequent comparisons between samples will evaluate the significance of intensity differences at any *m/z* in the elution profile (Finney et al. 2008; Meng et al. 2007). Advantages to this approach are the ability to measure differences without the use of stable isotope internal standards, a greater dynamic range for detection of differences, and the ability to measure differences between an unlimited number of aligned chromatograms. Finally, because chromatograms are aligned to one another, only a single MS/MS spectrum from any of the different samples (e.g., biological replicates or different time points) is needed to annotate the peptide identity of the difference region.

## Targeted Mass Spectrometry (or MS Westerns) for Hypothesis-Driven Proteomics (Quantitative Platform)

To address the limitations of sensitivity, selectivity, and throughput, there has been a recent shift toward the development and application of technologies for the targeted analysis of proteins within complex mixtures. Numerous derivations of targeted mass spectrometry using the specific acquisition of MS/MS spectra of peptides predicted in silico have been reported (Arnott et al. 2002; Chang et al. 2004), and more recently these methods have been based on the use of selected reaction monitoring (SRM) on triple quadrupole mass spectrometers (Anderson and Hunter 2006; Barnidge et al. 2004; Gerber et al. 2003). The concept of monitoring specific peptides from proteins of interest is well established. These methods have high specificity within a complex mixture and can be performed in a fraction of the instrument time compared with discovery-based methods. Ultimately, targeted studies are intended to complement discovery-based analyses and facilitate hypothesis-driven proteomic experiments.

**Figure 10:**
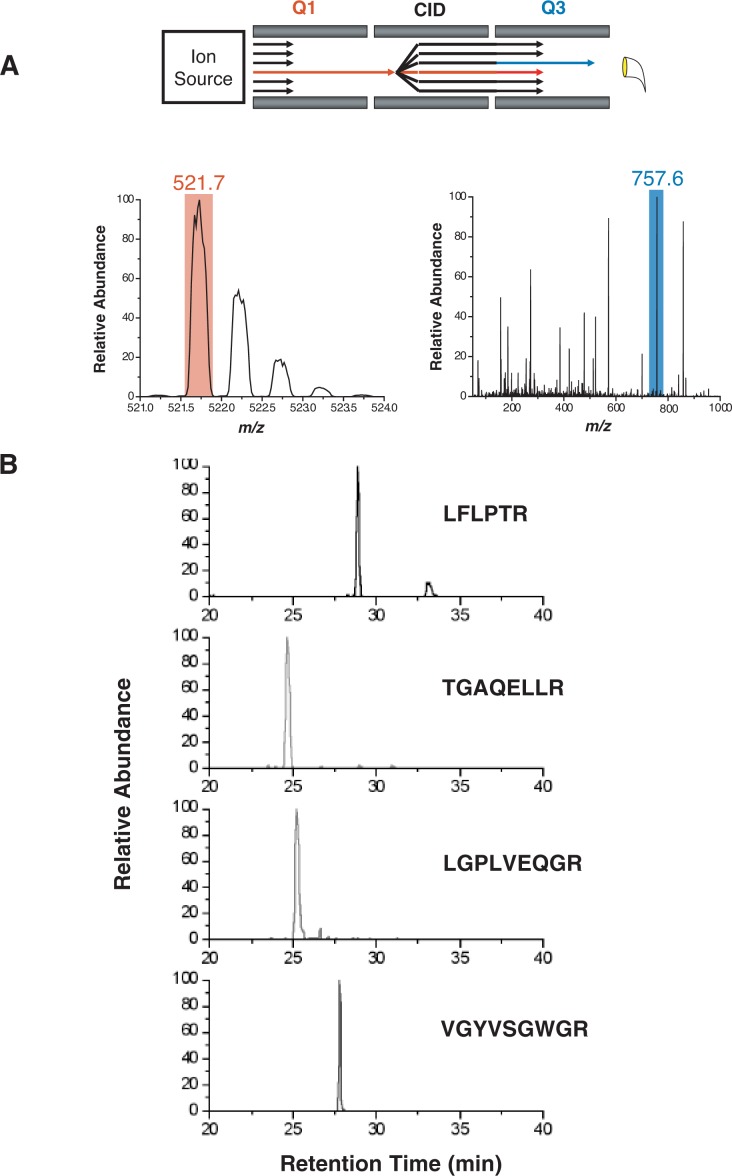
Illustration of selected reaction monitoring (SRM) on a triple quadrupole mass spectrometer. **A)** A predetermined precursor ion (mass-to-charge ratio, *m/z* 521.7) is selected in the first mass analyzer (Q1), fragmented by collision-induced dissociation (CID), and one of the resultant product ions (*m/z* 757.6) is selectively monitored in the second mass analyzer (Q3). **B)** Example of four different peptides generated by protein digestion with the enzyme trypsin (i.e., tryptic peptides) measured by SRM in human plasma.

Using the selectivity of multiple stages of mass selection of a tandem mass spectrometer (see figure 10), these targeted SRM assays are the mass spectrometry equivalent of a Western blot.^5^ In fact, Arnott and colleagues (2002) originally coined the term “MS Westerns” for the use of tandem mass spectrometry to target individual hypothesized peptides. Just as a Western blot only produces a signal for proteins within a complex mixture that are recognized by an antibody, a targeted mass spectrometry assay will only produce a signal for peptides that have a specific combination of precursor and product ion *m/z*. This combination of precursor and product ion *m/z* is extremely selective and referred to as a SRM transition. The advantage of using a targeted mass spectrometry–based assay is that it does not require creating any immunoaffinity reagents.

Targeted SRM assays can be developed for high-throughput assays that can measure multiple analytes in a single analysis (i.e., multiplexed quantitative assays) (Anderson and Hunter 2006). In a complex mixture, the chemical background of analytes that elute from a chromatography column at the same time (i.e., co-eluting analytes) can prohibit detection of a precursor ion in a data-dependent analysis. However, if the *m/z* of the precursor ion is known, a triple quadruple mass spectrometer can be used to minimize the chemical interference using two separate stages of mass analysis to selectively monitor a unique peptide. The combined specificity of chromatographic retention time, precursor ion mass, and product ion mass allow the selective detection of a unique peptide and its respective protein within a complex mixture. Importantly, by combining SRM with peptide standards of known quantities, absolute peptide amounts can be determined (Desiderio and Kai 1983; Desiderio et al. 1983; Gerber et al. 2003; MacCoss and Matthews 2005). Furthermore, a mass spectrometry–based assay facilitates the use of stable isotope–labeled analogs of proteins/peptides as internal standards that can account for errors in sample preparation or the mass spectrometric measurement. Targeted SRM assays can be multiplexed to quantify multiple transitions for multiple peptides in any given experiment and can be automated for high-throughput analyses.

## Conclusions and Future Directions

The proteomic analysis of brain tissue poses a complex analytical problem; this problem is further exacerbated in behavioral studies. However, given the throughput capability of differential mass spectrometry and the software available for the analysis of these data, combined with the sensitivity and multiplexing capability of targeted mass spectrometry, the promise of large-scale proteomics analyses in neuroscience is a certainty. As always, however, the “devil is in the details,” and although these technologies currently are possible, it will require continued application-driven developments in mass spectrometry hardware, sample preparation, automated sample handling, and computational analysis before these approaches become routine.

## Footnotes

1 For these strategies, animals are trained (i.e., conditioned) to seek or avoid alcohol under different experimental parameters. Researchers can then use consequences to modify the occurrence of the alcohol-seeking behavior.

2 For this fragmentation, peptides are collided with noble gases in the mass spectrometer to break them up into smaller partial peptides. If breaks occur randomly along the backbone of a peptide, the amino acid sequence of the peptide can be determined by finding the mass differences between the charged mass “ladders.”

3 Some mass spectrometers (e.g., linear ion trap mass spectrometers) can scan on the millisecond scale.

4 2D gel analysis (or electrophoresis) is used to separate proteins in an extract based on their electric charge (i.e., isoelectric point) and mass (i.e., molecular weight). Protein extracts typically are first applied to thin gel strips with a pH gradient and exposed to an electric current. Under the influence of this current, the proteins migrate through the gel strip, with the distance and direction traveled depending on the electric charge of the proteins. The gel strips then are loaded on a similar type of gel and exposed to a second electric current flowing in a direction perpendicular to the first one. Under these conditions, the proteins migrate from the initial gel strip into the second gel, with the distance traveled depending on the mass of the proteins. The proteins can then be made visible as “spots” on the gel through various staining procedures.

5 A Western blot (or immunoblot) is an analytical technique used to detect specific proteins in a given protein sample that involves three steps: (1) gel electrophoresis to resolve the proteins in the sample, (2) transfer of the proteins onto a membrane, and (3) detection of a selected protein using an immune molecule (i.e., antibody) that will bind specifically to that protein.
